# Effects of an Increased Financial Incentive on Follow-up in an Online, Automated Smoking Cessation Trial: A randomized Controlled Study Within a Trial

**DOI:** 10.1093/ntr/ntae068

**Published:** 2024-03-21

**Authors:** Juliet High, Kelly Grant, Aimie Hope, Lee Shepstone, Claire West, Antony Colles, Felix Naughton

**Affiliations:** Norwich Clinical Trials Unit, University of East Anglia, Norwich, UK; Norwich Clinical Trials Unit, University of East Anglia, Norwich, UK; Behavioural and Implementation Science Group, School of Health Sciences, University of East Anglia, Norwich, UK; Addiction Research Group, University of East Anglia, Norwich, UK; Norwich Clinical Trials Unit, University of East Anglia, Norwich, UK; Norwich Clinical Trials Unit, University of East Anglia, Norwich, UK; Norwich Clinical Trials Unit, University of East Anglia, Norwich, UK; Behavioural and Implementation Science Group, School of Health Sciences, University of East Anglia, Norwich, UK; Addiction Research Group, University of East Anglia, Norwich, UK

## Abstract

**Introduction:**

Poor retention in clinical trials can impact on statistical power, reliability, validity, and generalizability of findings and is a particular challenge in smoking cessation studies. In online trials with automated follow-up mechanisms, poor response also increases the resource need for manual follow-up. This study compared two financial incentives on response rates at 6 months follow up, in an online, automated smoking cessation feasibility trial of a cessation smartphone app (Quit Sense).

**Aims and Methods:**

A study within a trial (SWAT), embedded within a host randomized controlled trial. Host trial participants were randomized 1:1 to receive either a £10 or £20 voucher incentive, for completing the 6-month questionnaire. Stratification for randomization to the SWAT was by minimization to ensure an even split of host trial arm participants and by 6-week response rate. Outcome measures were: Questionnaire completion rate, time to completion, number of completers requiring manual follow-up, and completeness of responses.

**Results:**

Two hundred and four participants were randomized to the SWAT. The £20 and £10 incentives did not differ in completion rate at 6 months (79% vs. 74%; *p* = .362) but did reduce the proportion of participants requiring manual follow-up (46% vs. 62%; *p* = .018) and the median completion time (7 days vs. 15 days; *p* = .008). Measure response completeness rates were higher among £20 incentive participants, though differences were small for the host trial’s primary smoking outcome.

**Conclusions:**

Benefits to using relatively modest increases in incentive for online smoking cessation trials include more rapid completion of follow-up questionnaires and reduced manual follow-up.

**Implications:**

A modest increase in incentive (from £10 to £20) to promote the completion of follow-up questionnaires in online smoking cessation trials may not increase overall response rates but could lead to more rapid data collection, a reduced need for manual follow-up and reduced missing data among those who initiate completing a questionnaire. Such an improvement may help to reduce bias, increase validity and generalizability, and improve statistical power in smoking cessation trials.

**Trial Registration:**

Host trial ISRCTN12326962, SWAT repository store ID 164.

## Introduction

Poor retention of participants in clinical trials and the resulting missing outcome data are consistently documented challenges.^1,2^ The impacts include potential selection bias in results, reduced statistical power and reduced reliability, validity, and generalizability of the trial findings. Schultz et al.^3^ propose that a loss of greater than 20% potentially poses a threat to the validity of the study.^3^ However, all gains in collection of outcome data can have a meaningful impact^4^ so it is important that options are explored to improve retention in trials.

Retention of participants can be more challenging when trials are conducted remotely or wholly online^5^ and when there is minimal contact with participants due to non-interpersonal treatment delivery. For smoking cessation treatment, there are perceptions from participants that if they have not stopped smoking and their data are no longer useful, hence, the common application of a “missing = smoking” rule among those lost to follow-up.^6^ This further compounds the challenge of ensuring representative outcome data and retention rates in smoking cessation trials. Some online smoking cessation trials investigating remote treatments that have used incentives have achieved relatively high positive 6-month response rates, such as 77% of returning a saliva sample who received a £20 incentive,^7^ and 88% retained who received a $25 incentive plus a $10 bonus if done within 24 hours of notification.^8^ However, a Cochrane review (2017) of web-based smoking cessation trials identified that the majority of included studies achieved between 50% and 80% follow-up rates.^9^ The extent to which incentives promote response in smoking cessation trials remains largely unknown, particularly for remote and online trials.

Literature reviews investigating studies outside of smoking cessation find incentives to improve retention and response rates, particularly for remote trials.^10–12^ For example, Bailey et al.^13^ found postal response rates increased by 6%–10% through increasing the offered incentive from £10 to £20.

We embedded a randomized study within A trial (SWAT)^14^ in an online and automated feasibility randomized controlled trial of a stop-smoking smartphone app called Quit Sense, to understand the impact of offering a £20 incentive compared to a £10 incentive on: (1) participant response on their smoking status at 6 months follow-up by all methods, (2) the number of participants requiring manual follow up, (3) completeness of response from all follow-up questions asked. The trial results will inform which incentive rate to use in a future definitive trial of the Quit Sense app.

## Materials and Methods

### Design and Participants

A two-arm parallel randomized controlled study within a host trial^15^ was undertaken. The host trial recruited 209 patients to a randomized controlled trial comparing a Just-In-Time Adaptive Intervention smoking cessation smartphone app (Quit Sense), to online standard care for smoking cessation (NHS SmokeFree website) only.^16^ In the host trial, randomization was stratified by smoking rate (<16 vs. ≥ 16 cigarettes per day) and socioeconomic status (low vs. high). Recruitment, enrollment, baseline data collection, allocation, and intervention access delivery were automated through the study website. Outcomes were collected at 6 weeks and 6 months follow-up via the study website via a text message or email prompt or, if an initial response was not received, by telephone.

All participants of the host trial who had not withdrawn by the 6-week follow-up were randomized into the SWAT using minimization.^17^ Minimization ensured an equal balance between the SWAT (incentive) groups, accounting for the participants’ host trial allocation group, and whether they responded at 6-week follow-up. Allocation to the incentive groups was carried out in R using the minirand package, and then the results were imported into REDCap.^18,19^

The researcher was unaware of incentive group allocation when undertaking manual follow-up calls although was not blinded to incentive group as they needed to allocate vouchers when follow-up was completed using the REDCap database. Participants were blinded to their incentive allocation up until the incentive was offered at 6-month follow-up and were not informed at any point that the incentive amount offered was different for some participants.

Participants were offered a £5 Amazon gift voucher for completing the 6-week follow-up. Participants were informed in the participant information sheet they would receive a £10 voucher code for completing 6-month follow-up, but as the SWAT was conceived after all participants were recruited, they were not aware of the additional £10 incentive offered to some participants.

Additional consent to the SWAT was not sought as this was deemed unnecessary as all participants would still receive the £10 6-month incentive as indicated and informing the £10 incentive SWAT arm that they received less than £20 arm could have biased the findings. The SWAT was approved by the Wales NHS Research Ethics Committee 7 (19/WA/0361) as an amendment to the host trial ethics application.

The detailed plan for the SWAT and incentive rates were reviewed and agreed upon by a patient and public representative on the host trial management group and the SWAT was registered on the SWAT Repository Store (ID 164).

The sample size of this SWAT was dependent on the sample size used in the host trial, and so a formal power calculation was not undertaken.

Refer to the host trial outcomes paper^16^ for a description of host trial measures.

### Procedure

Six and a half months after enrollment (referred to as the 6-month follow-up, allowing 2 weeks for setting and preparing for a quit attempt), all participants were sent an automated text message inviting them to complete the final questionnaire. This message differed between groups only by the incentive amount they would receive (£10 or £20) (see Figure S1 for text message wording).

An automated reminder text message and a text message explaining the researchers would contact them by telephone if they did not complete the questionnaire online were sent 4 and 7 days later, respectively. Participants stating E-mail as a preferred contact method were sent the questionnaire link and reminders by E-mail instead, although very few did. Two weeks after the questionnaire’s due date, the Researcher attempted manual follow-up by telephone and, if unsuccessful, a final text message inviting them to respond to the primary smoking outcome question was sent.

Other strategies to try and improve overall response rates in the trial were used for all participants, including text reminders about participation and a “thank you” postcard.

### Analysis

Sample characteristics at baseline (by incentive arm) and completeness of responses are summarized, though no statistical comparisons were planned or undertaken. For the overall response rate analysis, a logistic regression model was run, using Wald confidence intervals for the odds ratio (OR), including stratification variables as covariates in the model and any participant characteristics that appeared imbalanced between the different incentive groups (prognostic variables), with response (binary) as the dependent variable. For the other analyses, further logistic regression models were run using an analogous approach for assessing the effect of incentives on manual follow-up rates. Results from regression analyses are presented separately as unadjusted, adjusted for stratification variables, and then adjusted for stratification and potential prognostic factors. Finally, a Cox proportional hazards analysis was undertaken with the log-rank test to assess the effects of incentives on completion time. Outcomes are described as proportions or summary statistics with 95% CIs.

## Results

Two hundred and four participants were included in the Quit Sense SWAT, 101 were randomized to the £20 incentive arm and 103 to the £10 arm.

The SWAT sample had a mean age of 41 years (range 18–61), 55% were female, 29% were in the lowest socioeconomic status category, 9% had other-than-White ethnicity and mean baseline smoking rate was 15 cigarettes per day (Table 1; Supplementary Table 2).

**Table 1.** Participant Characteristics at Baseline

	£20 incentive	£10 incentive	Overall
	(*n* = 101)	(*n* = 103)	(*n* = 204)
Host trial intervention group: *n* (%)	49 (48.5%)	51 (49.5%)	100 (49.0%)
Age at consent: mean (SD)	42.0 (9.6)	40.3 (10.6)	41.1 (10.2)
*Gender: n (%)*			
Male	51 (50.5%)	40 (38.8%)	91 (44.6%)
Female	50 (49.5%)	63 (61.1%)	113 (55.4%)
*Number of cigarettes smoked per day: n (%)*			
Less than 16	63 (62.4%)	62 (60.2%)	125 (61.3%)
16 or more	38 (37.6%)	41 (39.8%)	79 (38.7%)
Socioeconomic status^a^: *n* (%)			
Low	33 (32.7%)	26 (25.2%)	59 (28.9%)
High	68 (67.3%)	77 (74.8%)	145 (71.1%)
Missing	27	27	54
Ethnicity: *n* (%)			
White	91 (90.1%)	95 (92.2%)	186 (91.2%)
Indian	1 (1.0%)	0	1 (0.5%)
Pakistani	0	2 (1.9%)	2 (1.0%)
Bangladeshi	2 (2.0%)	1 (1.0%)	3 (1.5%)
Black African	0	2 (1.9%)	2 (1.0%)
Black (other)	1 (1.0%)	0	1 (0.5%)
Asian	3 (3.0%)	0	3 (1.5%)
Mixed race	2 (2.0%)	1 (1.0%)	3 (1.5%)
Not given	1 (1.0%)	2 (1.9%)	3 (1.5%)
Number of cigarettes smoked a day: mean (SD)	15.6 (7.1)	15.3 (6.8)	15.4 (6.9)
Employment status: *n* (%)			
* In work during last 12 months*	74 (73.3%)	75 (72.8%)	149 (73.0%)
Out of work for more than 12 months	26 (25.7%)	19 (18.5%)	45 (22.1%)
Retired	0	1 (1.0%)	1 (0.5%)
Time student	1 (1.0%)	8 (7.8%)	9 (4.4%)
Occupation: *n* (%)			
Modern professional	20 (27.0%)	17 (22.4%)	37 (24.7%)
Clerical	10 (13.5%)	8 (10.5%)	18 (12.0%)
Senior manager/administration	11 (14.9%)	11 (14.5%)	22 (14.7%)
Technical	10 (13.5%)	5 (6.6%)	15 (10.0%)
Semi routine manual/service	6 (8.1%)	11 (14.5%)	17 (11.3%)
Routine manual/service	5 (6.8%)	4 (5.3%)	9 (6.0%)
Middle/junior manager	8 (10.8%)	8 (10.5%)	16 (10.7%)
Traditional professional	4 (5.4%)	12 (15.8%)	16 (10.7%)
Missing	27	27	54
Highest qualification: *n* (%)			
No formal	9 (8.9%)	4 (3.9%)	13 (6.4%)
GCSE or similar	24 (23.8%)	18 (17.5%)	42 (20.6%)
A/AS Level or similar	28 (27.7%)	23 (22.3%)	51 (25.0%)
Degree or similar	36 (35.6%)	50 (48.5%)	86 (42.2%)
Other	4 (4.0%)	8 (7.8%)	12 (5.9%)

^a^Low socioeconomic status was classified as individuals with a semi-routine or routine and manual occupation (grade five in the National Statistics Socio-Economic Classification), or who have never worked or are long-term unemployed. High socioeconomic status included all those not classed as low status.

In the host trial, more participants provided data at 6 months than at 6 weeks. At 6 weeks, 149 (71%; 95% CI: 65%, 77%) were followed up and at 6 months, when the incentive rate was manipulated, this was 160 (77%; 95% CI: 71%, 82%). There were six host trial withdrawals, with five withdrawing prior to SWAT randomization (and so excluded) and one between 6 weeks and 6 months.

At 6-month follow-up, there was no statistically significant difference in overall response rate between participants offered a £20 voucher incentive compared with a £10 incentive (79% vs. 74%, unadjusted OR = 1.35; 95% CI: 0.71, 2.60; *p* = .36; fully adjusted OR = 1.29; 95% CI: 0.66, 2.54; *p* = .46; Table 2), though the results favored the £20 incentive group. However, the need for manual follow-up, due to questionnaires not being completed from digital prompts, differed between incentive groups; 46% of those in the £20 incentive group required manual follow-up compared with 62% of the £10 incentive group (unadjusted OR = 0.51, 95% CI: 0.29, 0.89, *p* = .018; fully adjusted OR = 0.53; 95% CI: 0.29, 0.95; *p* = .032).

**Table 2.** Effects of incentive value on response rate of the smoking status question at 6 months (self-reported prolonged abstinence), rate of manual follow up at 6 months and completion time (days) to completing the smoking status question at 6 months (in days)

Model^a^ *N* = 204	£20 incentive *N* = 101	£10 incentive *N* = 103	Odds ratio^b^	95% wald confidence Interval^c^	*p*-value^d^
*Overall response rate (n [%])*					
Incentive group only (unadjusted)	80 (79.2%)	76 (73.8%)	1.35	(0.71, 2.60)	.362
Adjusted model, including incentive group and stratification variables	80 (79.2%)	76 (73.8%)	1.38	(0.71, 2.69)	.337
Adjusted model, including incentive group, stratification, and prognostic variables	80 (79.2%)	76 (73.8%)	1.29	(0.66, 2.54)	.462
*Rate of manual follow-up (n [%])*					
Incentive group only (unadjusted)	46 (45.5%)	64 (62.1%)	0.51	(0.29, 0.89)	.018
Adjusted model, including incentive group and stratification variables	46 (45.5%)	64 (62.1%)	0.53	(0.30, 0.94)	.030
Adjusted model, including incentive group, stratification, and prognostic variables	46 (45.5%)	64 (62.1%)	0.53	(0.29, 0.95)	.032
*Number of days taken to respond (median [IQR])*					
Incentive group only (unadjusted)	7.00 (0.08, 18.13)	14.85 (0.15, 24.55)	1.55^e^	(1.12, 2.15)	.008
Adjusted model, including incentive group and stratification variables	7.00 (0.08, 18.13)	14.85 (0.15, 24.55)	1.53^e^	(1.10, 2.12)	.012
Adjusted model, including incentive group, stratification, and prognostic variables	7.00 (0.08, 18.13)	14.85 (0.15, 24.55)	1.53^e^	(1.10, 2.13)	.012

^a^Logistic regression model was used, modeling the odds of response to smoking status at 6 months (self-reported smoking status: where nonsmoking is smoking no more than 5 cigarettes within the 6-month study period) and modeling the odds of manual follow-up at 6 months. Both analyses adjusted for differences in number of cigarettes smoked per day at baseline, socioeconomic status at baseline (stratification variables), heaviness index at baseline, gender (prognostic variables), and incentive group.

^b^Odds ratio (OR; the odds of response/manual follow-up for participants in the £20 incentive group is (OR) times that of the odds of response for participants in the £10 incentive group).

^c^95% Wald confidence interval for odds ratio.

^d^The *p*-value is based upon a null hypothesis of zero difference.

^e^Hazard ratio (HR; at any particular point in time, the £20 incentive group are (HR) times more likely to respond to the 6-month questionnaire, compared to those participants in the £10 incentive group. If HR < 1 (or HR > 1), the £20 incentive group experiences a “risk” reduction (or “risk” increase) compared to the £10 incentive group).

The log-rank test, which compares expected and observed questionnaire completions over time by incentive group, shows moderate evidence that there was a difference in survival probability between incentive groups, with the £20 incentive group showing on average a lower completion time (median: 7.0 days, IQR 0.08, 18.13) than the £10 incentive group (median: 14.9 days, IQR 0.15, 24.55), which was statistically significant (unadjusted *p* = .008; Table 2). This is confirmed by a Cox proportional hazards analysis showing that at any point in time, the £20 incentive group was 55% more likely to respond to the 6-month questionnaire, compared to those participants in the £10 incentive group (unadjusted HR = 1.55, 95% CI: 1.12, 2.15, *p* = .008). The failure plot (Figure S2) suggests that failure probability increased over time, with a higher probability of completing the 6-month questionnaire when close to 0 days. Adjusting for stratification and prognostic variables for the above analyses did not alter the findings meaningfully.

Completeness of data for questionnaire items other than the overall response rate were also higher in the £20 incentive group compared to the £10 group (Table S1). For example, 78% versus 68% of participants reported urge items data and 78% versus 66% completed EQ-5D-5L questions in the £20 and £10 incentive groups respectively, though between-group differences for response to the primary outcome were smaller (79% vs. 74%).

## Discussion

Increasing the incentive for completing follow-up from £10 to £20 did not increase overall response rates at 6 months. However, it did reduce the proportion of participants requiring manual follow-up and the median completion time and may increase the completeness of some data. It is possible that incentives influence response to remote or non-interpersonal follow-up prompts more than response to interpersonal efforts. This is suggested by the beneficial effect of increased incentives on manual follow-up rates, where, if manual follow-up was not undertaken, a difference in overall response rates would potentially have been observed. Speeding up completion times could contribute to enhanced validity through a reduction in variability in response delay and would likely lead to other potential benefits such as a more rapid collection of saliva samples, when verifying those reporting abstinence.

If higher completeness of data are achieved, this would enhance statistical power and validity through reduced missing data. While higher incentives increase the overall incentive costs, these could be, at least in part, offset by reduced researcher costs with fewer participants to manually follow up. Furthermore, it is possible that an incentive higher than £20 may increase the response rate to a satisfactory level that would avoid the need for any manual follow-up, though further research would be needed to determine this, and the optimum level and type of incentive, noting that there is likely to be a ceiling effect.^20^ The overall response rate in the £20 incentive arm was very similar to another UK digital cessation intervention trial with a comparable incentive^7^ and the nonsignificant increase in response rates observed was in line with increased postal response rates found from increasing an incentive from £10 to £20 for an online intervention.^13^

This trial was automated, though where necessary participants were followed up by phone call. It is unclear whether the same incentive amounts would work as well in a different healthcare setting or population, particularly where motivations may be different. Response rates were in-line with or perhaps slightly higher than literature-reported rates for similar trials,^9^ indicating that appropriate incentives may help to achieve an acceptable response and retention rate.

Strengths of this SWAT include robust randomization and intervention delivery fidelity. Arms were well balanced for most baseline characteristics and key imbalances accounted for in the adjusted analyses. Participants were also effectively blinded from incentive allocation, so their response was likely to reflect the effect of the incentive within the context of the trial. However, it is possible some participants may have recalled the original incentive from the participant information sheet before the SWAT was embedded in the trial, and those offered £20 may have felt the increased incentive was a bonus or upgrade, which could have influenced the effects observed.

## Conclusion

While overall response rates were not increased, the potential benefit of a more rapid completion of follow-up questionnaires, a reduced need for manual follow-up, and potentially increased measure completion rates from a relatively modest increase in incentive costs may be worthwhile, particularly in the context of the high costs of running smoking cessation clinical trials and value of follow up data.

## Supplementary Material

ntae068_suppl_Supplementary_Tables_S1-S2_Figures_S1-S2

## Data Availability

The data underlying this article will be shared on reasonable request to the corresponding author.
