# Supplementary material for: Effects of an Increased Financial Incentive on Follow-up in an Online, Automated Smoking Cessation Trial: A randomized Controlled Study Within a Trial
Source: Nicotine Tob Res. 2024 Mar 21;26(9):1259–63. doi: 10.1093/ntr/ntae068 (PMC11339161; doi:10.1093/ntr/ntae068)
Supplement: ntae068_suppl_Supplementary_Tables_S1-S2_Figures_S1-S2 [file ntae068_suppl_supplementary_tables_s1-s2_figures_s1-s2.pdf]

Quit Sense incentive SWAT supplementary figures and tables

Supplementary Figure 1: wording of initial follow up prompt text message indicating incentive of either £10 or £20

> Hi [name]. Here's the final survey for Quit Sense: [survey-url] Your response is very valuable to this study. You can take part whatever stage you're at (smoking or quit) and it doesn't matter how involved you've felt with the study so far. Takes around 10 mins. £[10/20] Amazon voucher code on completion. We'll give you a call if we don't hear back, to see if you need any help.

Supplementary Figure 2: Failure plot for time taken to complete the 6-month follow up questionnaire by incentive arm

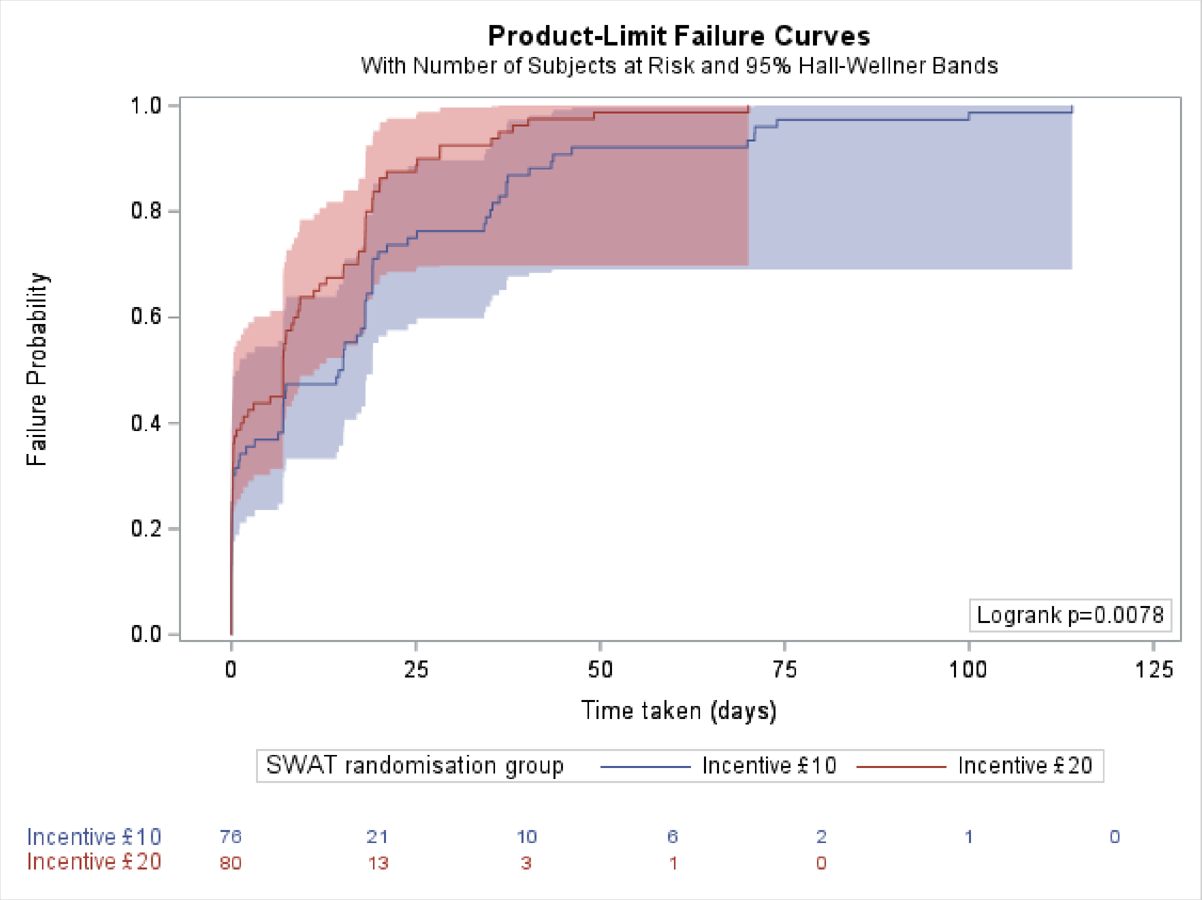

The plot shows the failure probability of 6-month follow up questionnaire completion by incentive group at multiple timepoints (1 minus the survival curve), giving the instantaneous failure rate. At the bottom, it shows the proportion of participants by each incentive group that are yet to complete the 6-month follow up questionnaire.

Supplementary Table 1: 6-month questionnaire response rates overall and by incentive group

6-month question	**£20 incentive proportion N=101**	**£10 incentive proportion N=103**	**Overall Proportion N=204**
Have you smoked any tobacco in the last 6 months?	79.2%	73.8%	76.5%
Are you currently smoking tobacco?	81.2%	73.8%	77.5%
How many cigarettes do you currently smoke? (of those reporting smoking tobacco)	96.2%	88.6%	92.7%
When did you last smoke a cigarette?	78.2%	68.9%	73.5%
How many serious quit attempts have you made during the study?	78.2%	68.0%	73.0%
Frequency of the urge to smoke (FUTS)	78.2%	68.0%	73.0%
Strength of the urge to smoke (SUTS):	78.2%	68.0%	73.0%
Smoking aid questions	78.2%	66.0%	72.1%
App questions (intervention group only)	75.5%	70.6%	73.0%
EQ5D-5L questions	78.2%	66.0%	72.1%
Self-efficacy questions	78.2%	67.0%	72.5%

Supplementary Table 2: Baseline outcome measures by SWAT group

	**£20 incentive (n=101)**	**£10 incentive (n=103)**	**Overall (n=204)**
Heaviness index: n (%)			
- Low	26 (25.7%)	37 (35.9%)	63 (30.9%)
- Moderate	67 (66.3%)	55 (53.4%)	122 (59.8%)
- High	8 (7.9%)	11 (10.7%)	19 (9.3%)
Frequency of the urge to smoke (FUTS): n (%)			
- A little of the time	10 (9.9%)	6 (5.8%)	16 (7.8%)
- Some of the time	28 (27.7%)	38 (36.9%)	66 (32.4%)
- A lot of the time	41 (40.6%)	43 (41.8%)	84 (41.2%)
- Almost all the time	14 (13.9%)	9 (8.7%)	23 (11.3%)
- All the time	8 (7.9%)	7 (6.8%)	15 (7.4%)
Strength of the urge to smoke (SUTS): n (%)			
- No urges	1 (1.0%)	0	1 (0.5%)
- Slight urges	6 (5.9%)	5 (4.9%)	11 (5.4%)
- Moderate urges	43 (42.6%)	39 (37.9%)	82 (40.2%)
- Strong urges	29 (28.7%)	38 (36.9%)	67 (32.8%)
- Very strong urges	16 (15.8%)	18 (17.5%)	34 (16.7%)
- Extremely strong urges	6 (5.9%)	3 (2.9%)	6 (5.9%)
EQ5D-5L utility score: mean (SD)	0.76 (0.25)	0.80 (0.18)	0.78 (0.21)
Missing	1	0	1
WISDM Automaticity subscale score: mean (SD)	4.92 (1.75)	4.59 (1.72)	4.75 (1.74)

WISDM Cue exposure/Associative processes subscale score: mean (SD)	4.48 (1.27)	4.74 (1.40)	4.61 (1.34)
Self-efficacy average score: mean (SD)	6.95 (3.46)	6.53 (3.52)	6.74 (3.49)
